# Alpha-Power Exponentiated Inverse Rayleigh distribution and its applications to real and simulated data

**DOI:** 10.1371/journal.pone.0245253

**Published:** 2021-01-14

**Authors:** Muhammad Ali, Alamgir Khalil, Muhammad Ijaz, Noor Saeed

**Affiliations:** Department of Statistics, University of Peshawar, Peshawar, Khyber Pakhtunkhwa, Pakistan; Tongii University, CHINA

## Abstract

The main goal of the current paper is to contribute to the existing literature of probability distributions. In this paper, a new probability distribution is generated by using the Alpha Power Family of distributions with the aim to model the data with non-monotonic failure rates and provides a better fit. The proposed distribution is called Alpha Power Exponentiated Inverse Rayleigh or in short APEIR distribution. Various statistical properties have been investigated including they are the order statistics, moments, residual life function, mean waiting time, quantiles, entropy, and stress-strength parameter. To estimate the parameters of the proposed distribution, the maximum likelihood method is employed. It has been proved theoretically that the proposed distribution provides a better fit to the data with monotonic as well as non-monotonic hazard rate shapes. Moreover, two real data sets are used to evaluate the significance and flexibility of the proposed distribution as compared to other probability distributions.

## Introduction

In statistical theory, the development of new distributions has become a common practice in recent decades; this is done generally by adding an extra parameter [1] to the baseline distribution, using generators [2, 3], or by combining two distributions [4]. Ramadan and Magdy [5] produced a new probability distribution by applying the Inverse Weibull (IW) to the Alpha Power Family of distribution. Alzaatreh et al. [2] introduced T-X family of continuous distributions by interchanging the probability density function of any continuous random variable with the probability density function of Beta distribution. Lee et al. [3] developed a technique of generating single variable continuous distributions. Jones [6] applied the Beta distribution to the family of distribution presented by Eugene et al. [7].

The main purpose of such an amendment to the existing distributions is to model the real data both with monotonically and non-monotonically hazard rate functions. Secondly, to increase the model flexibility of the complex data structures as compared to existing probability distributions. Because the existing distribution has some limitations so model the complex data structures, for example, Exponential and Weibull distributions fail the real data following a non-monotonic failure rate functions.

In this aim of presenting the paper is to contribute a new probability distribution that will model the data with both monotonically and non-monotonically hazard rate functions. The proposed model will also increase the model flexibility as compared to other models.

## Alpha Power Transformation

In the Recent past, Mahdavi and Kundu [8] suggested a new technique, called Alpha Power Transformation (APT), for including an additional parameter in the life time distributions. The primary purpose of this family was to utilize the non-symmetrical behavior of the parent distribution. The Alpha Power Transformation is defined by

Let *X* is a continuous random variable with *F(x)* as Cumulative Distribution Function, the Cumulative Distribution Function of Alpha Power Transformation is as follows;

$$F_{APT}(x)\;=\{\begin{array}{cc} \frac{\alpha^{F(x)}-1}{\alpha -1} & if\;\alpha >0,\;\;\alpha \neq 1\\ F(x) & if\alpha =1. \end{array}$$ (1)

The associated Probability density function is given below

$$f_{APT}(x)=\{\begin{array}{ll} \frac{\log\alpha }{\alpha -1}\alpha^{F(x)}f(x) & if\;\alpha >0,\;\;\alpha \neq 1\\ f(x) & if\alpha =1. \end{array}$$ (2)

The Alpha Power transformation has been used by many researchers, for example, Dey et.al [9] explored the new probability distributions by applying the Exponential and Rayleigh distribution to the Alpha Power Family of distributions. By using the same Transformation Nassar et al. [10] produced Alpha Power Weibull distribution, Alpha Power Inverse Weibull distribution was produced by Ramadan and Magdy [5], Alpha Power Transformed Extended Exponential distribution by Hassan et al [11].

The main aim of the paper is to produce a new probability distribution by using the Alpha Power family of distribution. In this paper, we considered the Exponentiated Inverse Rayleigh distribution as a baseline distribution presented by Rehman and Sajjad [12]. The Exponentiated Inverse Rayleigh distribution is the extension of the Inverse Rayleigh distribution presented by Voda [13]. He discussed various statistical properties such as moment generating function, survival function, and order statistics. A random variable X is said to be Inverse Rayleigh if it possesses the following Pdf and Cdf

$$f(y)=\frac{2\gamma }{y^{3}}e^{\frac{-\gamma }{y^{2}}};\;y>0,\gamma >0$$ (3)

$$F(y)=e^{\frac{-\gamma }{y^{2}}};\;y>0,\;\gamma >0.$$ (4)

The Exponentiated Inverse Rayleigh (EIR) distribution has the following pdf and cdf;

$$f(x)=\frac{2x\theta }{x^{3}}e^{\frac{-\alpha \theta }{x^{2}}};\;\;x,\;\alpha ,\;\theta >0$$ (5)

$$F(x)=e^{\frac{-\alpha \theta }{x^{2}}};\;x,\;\alpha ,\;\theta >0.$$ (6)

The current study is linked with the introduction of a novel distribution which is stated as Alpha Power Exponentiated Inverse Rayleigh (APEIR) distribution. Various statistical properties of the APEIR distribution are studied such as quantile function, median, mode, moment generating function and r^th^ moment, order statistics, mean residual life function, and stress strength parameter are obtained and discussed. Furthermore, an expression for the Renyi entropy and for the Mean Waiting Time has been explored. The estimation of the parameters is done by using the maximum likelihood. In order to prove the flexibility of the model, we considered the application by using two real data sets as well as simulated data.

## Alpha Power Exponentiated Inverse Rayleigh (APEIR) distribution

By applying the cumulative distribution function of the Exponentiated inverse Rayleigh distribution to the ALPF, we obtained the following Cdf and Pdf for the APEIR and is given by

$$F_{APEIR}(x)=\{\begin{array}{ll} \frac{\alpha^{e^{\frac{-\beta \theta }{x^{2}}}}-1}{\alpha -1} & \alpha >1\\ 0 & \alpha =1. \end{array}$$ (7)

$$f_{APEIR}(x)=\{\begin{array}{ll} \frac{log\alpha }{\alpha -1}\frac{2\beta \theta }{x^{3}}e^{\frac{-\beta \theta }{x^{2}}}\alpha^{e^{\frac{-\beta \theta }{x^{2}}}} & \alpha >1\\ f(x) & \alpha =1\\ 0 & otherwise \end{array}$$ (8)

**Fig 1:**
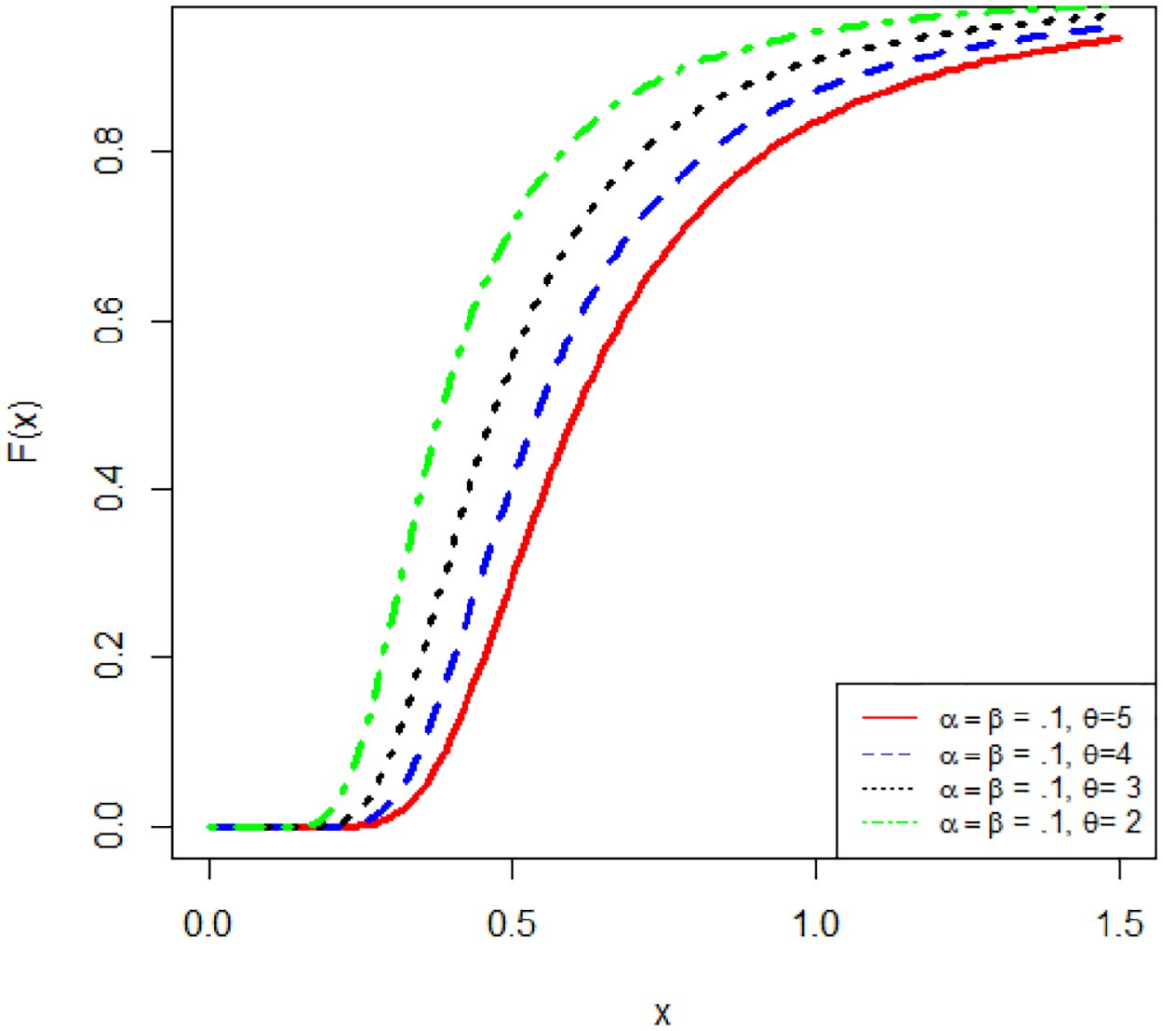
Graph of CDF of APEIR distribution.

Fig 1 reflects the graphical structure of the CDF of APEIR with various parameter values.

The hazard and survival function corresponding to the probability density function are as follows

$${{}_{\;}}_{\;}{{}_{\;}}_{\;}h_{APEIR}(x)=\{\begin{array}{c} \frac{2\beta \theta \;log\alpha \;e^{\frac{-\beta \theta }{x^{2}}}\;\;\alpha^{e^{\frac{-\beta \theta }{x^{2}}}}}{x^{3}(\alpha -\alpha^{e^{\frac{-\beta \theta }{x^{2}}}})}\;\;\;\alpha >1\;. \end{array}$$ (9)

$$S_{APEIR}(x)=\{\begin{array}{c} \frac{\alpha -\alpha^{e^{\frac{-\beta \theta }{x^{2}}}}}{\alpha -1}\;\;\;\;\;\;\;\;\alpha >1\;. \end{array}$$ (10)

**Fig 2:**
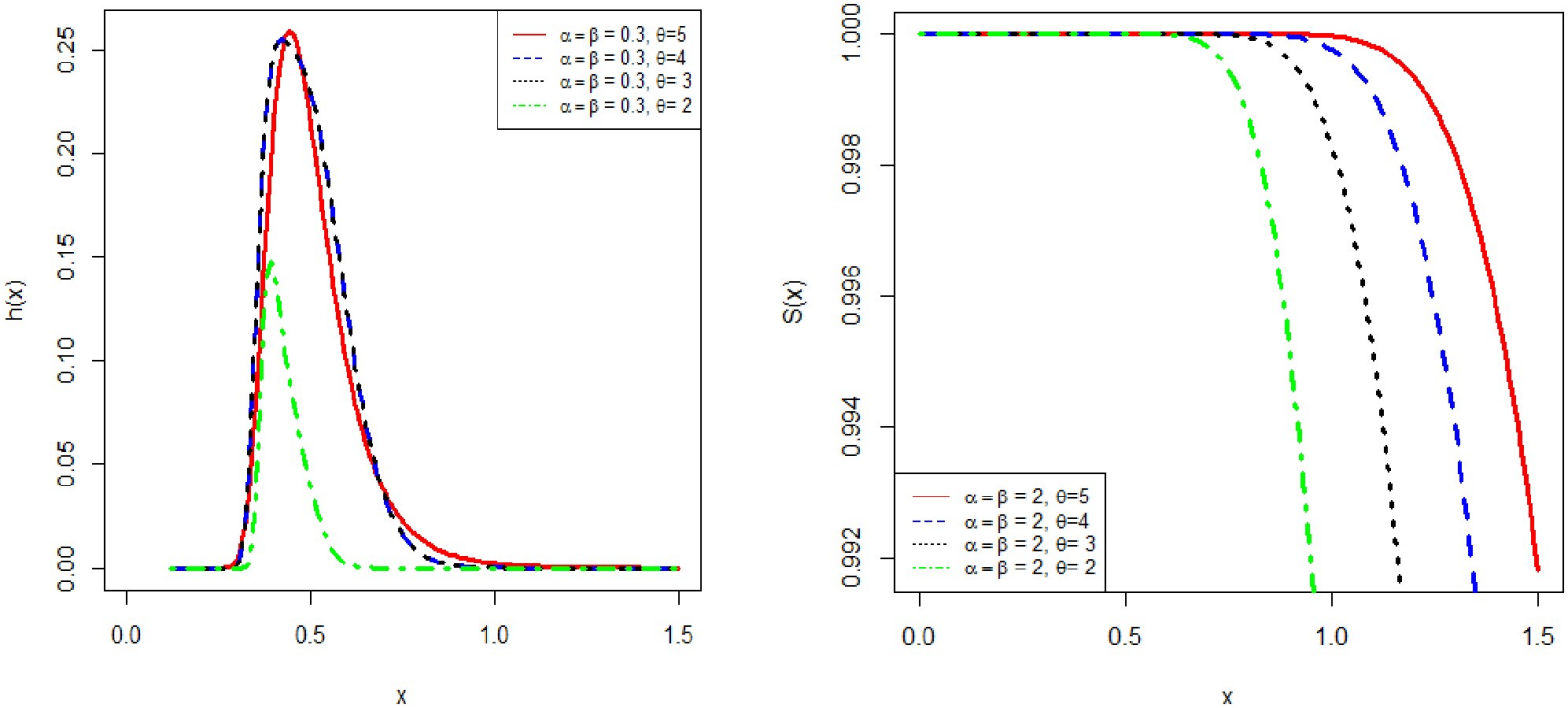
Graph of and hazard rate and survival function of APEIR distribution.

Fig 2 shows the hazard rate function and survival function of APEIR distribution with various values of parameters. Clearly, the hazard rate function of APEIR distribution is unimodal and positively skewed for *α* > 1.

**Lemma 1**: If *α* < 1 then *f*(*x*) is a decreasing function, this implies that *f*_*APEIR*_(*x*) is decreasing function.

**Proof:** If *f*(*x*) is a differentiable function and if its first order derivative or $\frac{d}{dx}logf(x)<0$ for *x in* (*α*, *β*, *θ*) then *f*(*x*) is a decreasing function and vice versa.

Taking the first derivative of *logf*_*APEIR*_(*x*) i.e.

$$\frac{d}{dx}logf_{APEIR}(x)=\frac{-3}{x}-\frac{2\beta \theta }{x^{3}}+\frac{2\beta \theta }{x^{3}}\;log\alpha \;e^{\frac{-\beta \theta }{x^{2}}}$$ (11)

For *α* < 1, *β and θ* > 0, which show that

$$\frac{d}{dx}logf_{APEIR}(x)<0,$$

Hence *f*_*APEIR*_(*x*) is a decreasing function.

**Lemma 2:** If *α* < 1 and *f*(*x*) is decreasing function so *f*(*x*) is log-convex hence *h*_*APEIR*_(*x*) is decreasing function.

**Proof:** If the second order derivative of *f*(*x*) exists and *f"*(*x*) > 0 or $\frac{d^{2}}{dx^{2}}logf(x)>0$, then *f*(*x*) is said to be log-convex.

Taking second order derivative of Eq (11), we get

$$\frac{d^{2}}{dx^{2}}logf_{APEIR}(x)=\frac{-3}{x}-\frac{6\beta \theta }{x^{4}}-\frac{6\beta \theta }{x^{4}}\;log\alpha \;e^{\frac{-\beta \theta }{x^{2}}}+\;\frac{4\beta^{2}\theta^{2}}{x^{6}}\;log\alpha \;e^{\frac{-\beta \theta }{x^{2}}},$$ (12)

0 < *α* < 1, *β and θ >* 0

Then $\frac{d^{2}}{dx^{2}}logf_{APEIR}(x)>0$.

Therefore *f*_*APEIR*_(*x*) is log-convex.

### Quantile function

Let X ∼APEIR (α β, θ) then its Quantile function is given below;

$F(X)\;=u\;which\;impliesX=F^{-1}(u),$ where *u* is uniformly distributed. The Quantile function of APEIR distribution is

$$x_{p}={\left[\frac{-\beta \theta }{\text{log}\{\frac{\text{log}\{u(\alpha -1)+1\}}{\text{log}\alpha }\}}\right]}^{\frac{1}{2}}.$$ (13)

Median of APEIR distribution is obtained by substituting u = 1/2 in Eq (13), we get

$$Median={\left[\frac{-\beta \theta }{log\{\frac{log\{\frac{1}{2}(\alpha +1)\}}{log\alpha }\}}\right]}^{\frac{1}{2}}.$$ (14)

### Mode

Mode of APEIR distribution is that point by which the distribution reaches its maximum point and it is obtained by solving the following equation

$$\frac{d}{dx}f_{APEIR}(x)=0$$

$$\frac{d}{dx}[\frac{log\alpha }{\alpha -1}\;\frac{2\beta \theta }{x^{3}}\;e^{\frac{-\beta \theta }{x^{2}}}\alpha^{e^{\frac{-\beta \theta }{x^{2}}}}]=0$$

We finally, obtained the result

$$x={\left[\frac{3}{-2\beta \theta (1+log\alpha )}\right]}^{-1/2}.$$ (15)

### Rth moment of APEIR distribution

Let X ∼APEIR (α β, θ), then the expression of its *r*^*th*^ moment is as follows;

$$\mu^{/}{}_{r}={\;\text{E(X}}^{r})=\int_{0}^{\infty }x^{r}\;\frac{log\alpha }{\alpha -1}\;\frac{2\beta \theta }{x^{3}}\;e^{\frac{-\beta \theta }{x^{2}}}\;\alpha^{e^{\frac{-\beta \theta }{x^{2}}}}dx^{\;},$$ (16)

Using *y* = *x*^−2^ and series notation $\;\alpha^{-z}=\sum_{k=0}^{\infty }\frac{{\left(-log\alpha \right)}^{k}}{k!}{\left(-z\right)}^{k}$ and $\text{log}z=-\sum_{j=1}^{\infty }\frac{{\left(-1\right)}^{j}{\left(-1+z\right)}^{j}}{j}$, we get the final result as

$$\mu^{/}{}_{r}=\;{\left(\frac{-1}{\beta \theta }\right)}^{l}\frac{log\alpha }{\alpha -1}\sum_{k=o}^{\infty }\sum_{i=0}^{jl}\;\overset{\infty }{\underset{j=1}{\sum }}\;\frac{{\left(-1\right)}^{jl+l+i+2k}}{j^{l}}\;(\begin{array}{c} jl\\ i \end{array})\;\frac{{\left(log\alpha \right)}^{k}}{k!}\;\frac{1}{\left(jl-i+k+1\right)}.$$ (17)

where $l=\frac{-r}{2}$. which is the required result.

### Moment Generating Function (MGF)

Let *X* ~ *APEIR*(*α*, *β*, *θ*) then the expression for its MGF is as follows;

$$M_{x}(t)=\;E\;(\;e^{tx})=\int_{0}^{\infty }e^{tx}\frac{log\alpha }{\alpha -1}\;\frac{2\beta \theta }{X^{3}}\;e^{\frac{-\beta \theta }{x^{2}}}\;\alpha^{e^{\frac{-\beta \theta }{x^{2}}}}dx,$$ (18)

Using *y* = *x*^−2^, $e^{tx}=\sum_{r=0}^{\infty }\frac{t^{r}x^{r}}{r!}$ and the series representation $\alpha^{-z}=\sum_{k=0}^{\infty }\frac{{\left(-log\alpha \right)}^{k}}{k!}{\left(-z\right)}^{k}$ in Eq (18).

The MGF of APEIR distribution has the following form

$$M_{X}(t)=\;{\left(\frac{-1}{\beta \theta }\right)}^{l}\frac{log\alpha }{\alpha -1}\sum_{r=0}^{\infty }\sum_{k=0}^{\infty }\sum_{i=0}^{jl}\overset{\infty }{\underset{j=1}{\sum }}\frac{t^{r}}{r!}\;\frac{{\left(-1\right)}^{jl+l+i+2k}}{j^{l}}\;(\begin{array}{c} jl\\ i \end{array})\;\frac{{\left(log\alpha \right)}^{k}}{k!}\;\frac{1}{\left(jl-i+k+1\right)}.$$ (19)

where $l=\frac{-r}{2}$.

### Mean residual life function

Let X be the survival time of an object having pdf “f(x)” and survival function specified in Eq (10), the mean residual life function is the average remaining lifespan, which is a component survived up to time t. The mean residual life function, say, *μ*(*t*) has the following expression.

$$\mu (t)=\frac{1}{P(X>t)}\int_{t}^{\infty }P(X>x)dx\;,\;\;\;\;\;t\geq 0$$

$$\mu (t)=\frac{1}{S(t)}(-\int_{0}^{t}xf(x)dx+E(t))-t,\;t\geq 0$$ (20)

where

$$\int_{o}^{t}xf(x)dx={\left(\frac{-1}{\beta \theta }\right)}^{\frac{-1}{2}}\frac{log\alpha }{\alpha -1}\overset{\infty }{\underset{j=1}{\sum }}\sum_{i=0}^{jl}\overset{\infty }{\underset{k=0}{\sum }}\frac{{\left(-1\right)}^{jl+l+i+2k}}{j^{l}}\;(\begin{array}{c} jl\\ i \end{array})\;\frac{{\left(log\alpha \right)}^{k}}{k!}\;\frac{e^{\frac{-\beta \theta (jl-i+k+1)}{t^{2}}}}{jl-i+k+1},$$ (21)

and

$$E(t)=\frac{1}{\theta }{\left(\frac{-1}{\beta \theta }\right)}^{\frac{-1}{2}}\frac{log\alpha }{\alpha -1}\overset{\infty }{\underset{j=1}{\sum }}\sum_{i=0}^{jl}\overset{\infty }{\underset{k=0}{\sum }}\frac{{\left(-1\right)}^{jl+l+i+2k}}{j^{l}}\;(\begin{array}{c} jl\\ i \end{array})\;\frac{{\left(log\alpha \right)}^{k}}{k!}\;\frac{1}{jl-i+k+1},$$ (22)

putting Eqs (10), (21) and (22) in Eq (20), we get

$$\mu (t)=\frac{\begin{array}{l} \left(\alpha -1){\left(\frac{-1}{\beta \theta }\right)}^{l}\;\frac{log\alpha }{\left(\alpha -1\right)}\;\;\overset{\infty }{\underset{j=1}{\sum }}\sum_{i=0}^{jl}\overset{\infty }{\underset{k=0}{\sum }}\frac{{\left(-1\right)}^{jl+l+i+2k}}{j^{l}}\;(\begin{array}{c} jl\\ i \end{array})\;\frac{{\left(log\alpha \right)}^{k}}{k!}\;\{\;\frac{1-\theta e^{\frac{-\beta \theta (jl-i+k+1)}{t^{2}}}}{\theta (jl-i+k+1)}\right)\\ -t(\alpha -\alpha^{e^{\frac{-\beta \theta }{x^{2}}}}) \end{array}}{\left(\alpha -\alpha^{e^{\frac{-\beta \theta }{x^{2}}}}\right)}$$ (23)

where $l=\frac{-1}{2}$.

### Order statistics

Let *X*_1_, *X*_2_, *X*_3_, …, *X*_*n*_ be a random sample of size n from APEIR distribution and let *X*_(1)_ ≤ *X*_(2)_ ≤ … ≤ *X*_(*n*)_ denote the order statistics. Let *X*_*i*:*n*_ denotes the *i*^*th*^ order statistics, then the Probability Density function of *X*_*i*:*n*_ is given by

$$\;\;\;f_{i:n}(x)=\frac{n!}{(i-1)!(n-i)!}f(x)\;{\left[F(x)\right]}^{i-1}\;{\left[1-F(x)\right]}^{n-i},$$ (24)

putting Eqs (7) and (8) of APEIR distribution in (24), we obtain the pdf of *i*^*th*^ order statistic for *x* > 0, as is mentioned below

$$f_{i:n}(x)=\frac{n!}{(i-1)!(n-i)!}\frac{\text{log}\alpha }{{\left(\alpha -1\right)}^{n}}\;\frac{2\beta \theta }{x^{3}}\;e^{\frac{-\beta \theta }{x^{2}}}\;\alpha^{e^{\frac{-\beta \theta }{x^{2}}}}{\left[\alpha^{e^{\frac{-\beta \theta }{x^{2}}}}-1\right]}^{i-1}{\left[\alpha -\alpha^{e^{\frac{-\beta \theta }{x^{2}}}}\right]}^{n-i},$$ (25)

by inserting *i* = 1 in Eq (25), we obtain the smallest order statistic as follows:

$$f_{1:n}(\text{x})=\frac{2n\beta \theta log\alpha }{{\left(\alpha -1\right)}^{n}}\frac{1}{x^{3}}\;e^{\frac{-\beta \theta }{x^{2}}}\alpha^{e^{\frac{-\beta \theta }{x^{2}}}}{\left[\alpha -\alpha^{e^{\frac{-\beta \theta }{x^{2}}}}\right]}^{n-1}.$$ (26)

For largest order statistic insert i = n in Eq (25), we get

$$\;\;\;f_{n:n}(\text{x})=\frac{n\;log\alpha }{{\left(\alpha -1\right)}^{n}}\;\frac{2\beta \theta }{x^{3}}\;e^{\frac{-\beta \theta }{x^{2}}}\;\alpha^{e^{\frac{-\beta \theta }{x^{2}}}}{\left[\alpha^{e^{\frac{-\beta \theta }{x^{2}}}}-1\right]}^{n-1}.$$ (27)

Put *i* = *n* /2 in Eq (25), to obtain the distribution of median, we have

$$f_{\frac{n}{2:}:n}(\text{x})=\;\frac{n!\;log\alpha }{{\left(\alpha -1\right)}^{n}}\;\frac{2\beta \theta }{x^{3}}\;\frac{1}{(\frac{n}{2}-1)!\;(n-\frac{n}{2})!}\;e^{\frac{-\beta \theta }{x^{2}}}\;\alpha^{e^{\frac{-\beta \theta }{x^{2}}}}{\left[\alpha^{e^{\frac{-\beta \theta }{x^{2}}}}-1\right]}^{\frac{n}{2}-1}{\left[\alpha -\alpha^{e^{\frac{-\beta \theta }{x^{2}}}}\right]}^{n-\frac{n}{2}}.$$ (28)

### Stress-strength parameter

Let *X*_1_, *X*_2_ are independently and identically distributed variables such that *X*_1_ ~ *APEIR*(*α*_1_, *θ*_1_, *β*) and *X*_2_ ~ *APEIR*(*α*_2_, *θ*_2_, *β*) then its stress strength parameter has the following expression.

$$R=\int_{-\infty }^{\infty }f_{1}(x)F_{2}(x)dx.$$

using Eqs (7) and (8) of APEIR distribution then Stress Strength Parameter is given as;

$$R=\frac{2\beta \theta_{1}log\alpha_{1}}{\left(\alpha_{1}-1)(\alpha_{2}-1\right)}\int_{0}^{\infty }x^{-3}\;e^{\frac{-\beta \theta_{{}_{1}}}{x^{2}}}\;\alpha_{1}{}^{e^{\frac{-\beta \theta_{1}}{x^{2}}}}\;\alpha_{2}{}^{e^{\frac{-\beta \theta_{2}}{x^{2}}}}\;dx-\;\frac{1}{\left(\alpha_{2}-1\right)},$$ (29)

after simplification, we finally obtained the equation for Stress-Strength Parameter.

$$\;R=\frac{\beta \theta_{1}\text{lo}g\alpha_{1}}{\left(\alpha_{1}-1)(\alpha_{2}-1\right)}\overset{\infty }{\underset{k=0}{\sum }}\frac{{\left(log\alpha_{1}\right)}^{k}(\;log\alpha_{2)}{}^{m}\;{\left(-1\right)}^{2k+2m}}{k!m!}\;\frac{1}{\left(\beta \theta_{1}+\beta \theta_{1}k+\beta \;\theta_{2}m\right)}-\;\frac{1}{\left(\alpha_{1}-1\right)}\;.$$ (30)

**Lemma 3:** Let *X*~ *APEIR*(*α*, *θ*, *β*), then its Renyi entropy is defined by

$$RE_{x}(v)=\frac{1}{1-v}log[\frac{\beta log\alpha }{\alpha -1}]{\left(-v\beta \theta \right)}^{-l}\overset{\infty }{\underset{j=1}{\sum }}\sum_{i=0}^{jl}\sum_{k=0}^{\infty }\frac{{\left(-1\right)}^{jl+l+i+2k}}{j^{l}}\;(\begin{array}{c} jl\\ i \end{array})\frac{{\left(log\alpha \right)}^{k}}{k!\;(jl-i+k+1)}.$$ (31)

where $l=\frac{3(v-1)}{2}.$

**Proof:** For APEIR distribution, Renyi entropy has the following expression;

$$\begin{array}{l} RE_{X}(v)=\frac{1}{1-v}\text{log}\{\int_{-\infty }^{\infty }f{\left(x\right)}^{v}dx)\\ =\frac{1}{1-v}\int_{0}^{\infty }{\left(\frac{log\alpha }{\alpha -1}\;\frac{2\beta \theta }{x^{3}}\;e^{\frac{-\beta \theta }{x^{2}}}\alpha^{e^{\frac{-\beta \theta }{x^{2}}}}\right)}^{v}dx \end{array}$$

The result can be obtained easily by substituting $\alpha^{-z}=\sum_{k=0}^{\infty }\frac{{\left(-log\alpha \right)}^{k}}{k!}{\left(-z\right)}^{k}.$

**Lemma4:** The Mean Waiting Time say $\bar{\mu }(t)$ of APEIR distribution is as follows;

$$\bar{\mu }(t)=\frac{t(\alpha^{e^{\frac{-\beta \theta }{t^{2}}}}-1)-(\alpha -1)[{\left(\frac{-1}{\beta \theta }\right)}^{-1/2}\;\frac{log\alpha }{\left(\alpha -1\right)}\;\;\overset{\infty }{\underset{j=1}{\sum }}\sum_{i=0}^{jl}\overset{\infty }{\underset{k=0}{\sum }}\frac{{\left(-1\right)}^{jl+l+i+2k}}{j^{l}}\;\;\frac{{\left(log\alpha \right)}^{k}}{k!}\;\{\frac{e^{\frac{-\beta \theta (jl-i+k+1)}{t^{2}}}}{\left(jl-i+k+1\right)}\}]}{\left(\alpha^{e^{\frac{-\beta \theta }{t^{2}}}}-1\right)}.$$ (32)

**Proof:** For APEIR, the mean waiting time is given by

$$\bar{\mu }(t)=t-\frac{1}{F(t)}\int_{0}^{\infty }x\;f(x)dx,$$

the result can be obtained easily by substituting $\alpha^{-z}=\sum_{k=0}^{\infty }\frac{{\left(-log\alpha \right)}^{k}}{k!}{\left(-z\right)}^{k}.$

### Parameters estimation

Let we have a random sample of size “n” from APEIR (*α*, *β*, *θ*), then their joint density function is as follows;

$$l(\alpha ,\beta ,\theta )=(\frac{log\alpha }{\alpha -1}){\;}^{n}{\left(2\beta \theta \right)}^{n}\;\frac{1}{\prod_{i=1}^{n}x_{i}{}^{3}}\;e^{-\beta \theta \sum_{i=1}^{n}\frac{1}{x^{2}{}_{i}}}-\alpha^{\sum_{i=1}^{n}e^{\frac{\beta \theta }{x^{2}{}_{i}}}},$$ (33)

taking the logarithm, Eq (33) becomes

$$logl(\alpha ,\beta ,\theta )=nlog(\frac{log\alpha }{\alpha -1})+nlog(2\beta \theta )-\text{log}(\prod_{i=1}^{n}x_{i}{}^{3})-\beta \theta \;\sum_{i=1}^{n}\frac{1}{x^{2}{}_{i}}-\sum_{i=1}^{n}e^{\frac{\beta \theta }{x^{2}{}_{i}}}\;log\alpha ,$$ (34)

differentiating Eq (34) with respect to *α*, *β* and *θ*, and taking equal to 0, we get the following normal equations;

$$\frac{\partial logl(\alpha ,\beta ,\theta )}{\partial \alpha }=\frac{n(\alpha -1-log\alpha )}{\alpha (\alpha -1)log\alpha }-\frac{1}{\alpha }\sum_{i=1}^{n}e^{\frac{\beta \theta }{x^{2}{}_{i}}}\;=0,$$ (35)

$$\frac{\partial logl(\alpha ,\beta ,\theta )}{\partial \beta }=\frac{n}{\beta }-\theta \;\sum_{i=1}^{n}\frac{1}{x^{2}{}_{i}}-\sum_{i=1}^{n}e^{\frac{\beta \theta }{x^{2}{}_{i}}}\;(\frac{\theta }{x^{2}{}_{i}})log\alpha =0,$$ (36)

$$\frac{\partial logl(\alpha ,\beta ,\theta )}{\partial \theta }=\frac{n}{\theta }-\;\beta \;\sum_{i=1}^{n}\frac{1}{x^{2}{}_{i}}-\sum_{i=1}^{n}e^{\frac{\beta \theta }{x^{2}{}_{i}}}\;(\frac{\beta }{x^{2}{}_{i}})log\alpha =0.$$ (37)

By solving (35), (36) and (37) all together, we get the estimates of *α*, *β* and *θ*. We can get the solution of the above equations by using methods like Newton Raphson method or Bisection method. ML Estimators follows asymptotically normally distribution, that is $\sqrt{n}(\hat{\alpha }-\alpha ,\hat{\beta }-\beta ,\hat{\theta }-\theta )\sim N_{3}(0,\Sigma )$, ∑ is a matrix contains variability measures of the estimated parameters and computed from the following F matrix;

$$F=[\begin{array}{ccc} \frac{\partial^{2}logl}{\partial \alpha^{2}} & \frac{\partial^{2}logl}{\partial \alpha \partial \beta } & \frac{\partial^{2}logl}{\partial \alpha \partial \theta }\\ \frac{\partial^{2}logl}{\partial \beta \partial \alpha } & \frac{\partial^{2}logl}{\partial \beta^{2}} & \frac{\partial^{2}logl}{\partial \beta \partial \theta }\\ \frac{\partial^{2}logl}{\partial \theta \partial \alpha } & \frac{\partial^{2}logl}{\partial \theta \partial \beta } & \frac{\partial^{2}logl}{\partial \theta^{2}} \end{array}],$$

again, differentiating Eqs (35), (36) and (37) w.r.t *α*, *β and θ*, we obtained;

$$\frac{\partial^{2}logl}{\partial \alpha^{2}}=n[\frac{\alpha (\alpha -1)log\alpha (1-\frac{1}{\alpha })-\{\left(\alpha -1-log\alpha )((\alpha -1)+(2\alpha -1)log\alpha \right)\}}{{\left(\alpha (\alpha -1)log\alpha \right)}^{2}})+\frac{1}{\alpha^{2}}\;\sum_{i=1}^{n}e^{\frac{\beta \theta }{x^{2}{}_{i}}}$$ (38)

$$\frac{\partial^{2}logl}{\partial \beta^{2}}=-\frac{n}{\beta^{2}}-\theta^{2}log\alpha \sum_{i=1}^{n}x_{i}{}^{2}e^{\frac{\beta \theta }{x^{2}{}_{i}}}\;.$$ (39)

$$\frac{\partial^{2}logl}{\partial \theta^{2}}=-\frac{n}{\theta^{2}}-\beta^{2}log\alpha \sum_{i=1}^{n}e^{\frac{\beta \theta }{x^{2}{}_{i}}}\;\frac{1}{x_{i}{}^{4}}\;.$$ (40)

$$\frac{\partial^{2}logl}{\partial \alpha \partial \beta }=-\frac{\sum_{i=1}^{n}e^{\frac{\beta \theta }{x^{2}{}_{i}}}}{\alpha }\;\frac{\theta }{x^{2}{}_{i}}.$$ (41)

$$\frac{\partial^{2}logl}{\partial \alpha \partial \theta }=-\frac{\sum_{i=1}^{n}e^{\frac{\beta \theta }{x^{2}{}_{i}}}}{\alpha }\;\frac{\beta }{x^{2}{}_{i}}.$$ (42)

$$\frac{\partial^{2}logl}{\partial \beta \partial \theta }=-\sum_{i=1}^{n}\frac{1}{x^{2}{}_{i}}-log\alpha \sum_{i=1}^{n}e^{\frac{\beta \theta }{x^{2}{}_{i}}}\;\frac{\theta^{2}}{x^{4}{}_{i}}.$$ (43)

Large sample (1 − ζ)100% confidence interval for the suggested distribution parameters has the following expression;

$$\hat{\alpha }\pm Z_{\zeta /2}\sqrt{\Sigma_{11}}.$$

$$\hat{\beta }\pm Z_{\zeta /2}\sqrt{\Sigma_{22}}.$$

$$\hat{\theta }\pm Z_{\zeta /2}\sqrt{\Sigma_{33}}.$$

### Simulations study

The parameter estimates of APEIR distribution, their Mean Square Error (MSE) as well as the bias measure are computed using a simulation study with 1000 replications each with a sample of size *n* = 30, 70, 130 *and* 170. A simulated data is generated from APEIR distribution using the following expression

$$X={\left[\frac{-\beta \theta }{log\{\frac{log\{u(\alpha -1)+1)}{log\alpha }\}}\right]}^{\frac{1}{2}},$$

where *U* follows a standard uniform distribution. The average bias and MSE are computed by using the mathematical formulae as under

$$Bias=\frac{1}{W}\overset{w}{\underset{1=1}{\sum }}(\hat{b_{i}}-b)$$

$$MSE=\frac{1}{W}\overset{w}{\underset{1=1}{\sum }}{\left(\hat{b_{i}}-b\right)}^{2}$$

where *b* = (*α*, *β*, *θ*). The average bias and MSEs are given in Table 1. It has been observed that the MSEs and bias of the estimates are decreasing for all parameter combinations with the increase in the sample of size n.

**Table 1:** Average values of MLE, corresponding MSE and bias.

Parameter	n	MSE ($\hat{\alpha }$)	MSE ($\hat{\beta }$)	MSE ($\hat{\theta }$)	Bias ($\hat{\alpha }$)	Bias ($\hat{\beta }$)	Bias ($\hat{\theta }$)
α = 0.5	30	1.600291	0.140596	0.076240	0.296260	0.034060	0.029888
β = 1.5	70	0.274669	0.080418	0.044591	0.093877	0.014399	0.012343
θ = 2	130	0.148004	0.078995	0.043825	0.060626	0.004091	0.003544
	170	0.079246	0.030490	0.017141	0.042915	0.003248	0.001590
α = 1.5	30	3.781425	0.234186	0.153185	0.367721	0.132582	0.112368
β = 2	70	3.080643	0.101876	0.066932	0.338951	0.062667	0.052157
θ = 2.5	130	1.926952	0.067971	0.038254	0.278251	0.026125	0.022521
	170	0.741660	0.052949	0.037985	0.115542	0.021330	0.018270
α = 0.5	30	1.088054	0.066637	0.031088	0.231466	0.029975	0.022030
β = 1	70	0.459863	0.038682	0.008550	0.104862	0.018523	0.013168
θ = 1.5	130	0.180847	0.027694	0.006936	0.063190	0.003021	0.002425
	170	0.163698	0.015616	0.004694	0.049297	0.002392	0.000973
α = 0.5	30	1.614823	0.195070	0.138455	0.306416	0.068536	0.062257
β = 2.5	70	0.351019	0.114477	0.079823	0.104602	0.032169	0.028678
θ = 3	130	0.179059	0.107070	0.078002	0.064438	0.006292	0.005975
	170	0.163570	0.066727	0.046419	0.051060	0.006192	0.005712

### Applications

In this section, we provide two applications of the proposed distribution to the lifetime data. The performance of the suggested model is checked by the goodness of fit criteria including they are the AIC, CAIC, BIC, HQIC, and the P-value. For more details of the goodness of fit criteria, we refer to see [14–19]. In general, with fever values of these statistics, a probability model would perform better than others. The proposed model is compared with Exponentiated Inverse Rayleigh distribution by Rehman and Sajjad [12], Weibull Rayleigh distribution by Merovci and Elbatal [20], Generalized Rayleigh distribution by Raqab and Madi [21], two parameter Rayleigh distribution by Dey et.al [22], Transmuted inverse Rayleigh distribution by Afaq et al [23] and modified inverse Rayleigh distribution by Muhammad [24]. The probability functions of these distributions are given by

- Exponentiated Inverse Rayleigh Distribution
  $$f(x)=\frac{2\beta \theta }{x^{3}}\;e^{\frac{-\beta \theta }{x^{2}}}\theta ,\;\;\beta ,\;X>0.$$
- Weibull Rayleigh (WR) Distribution
  $$f(x)=\alpha \beta \theta xe^{\frac{\theta x^{2}}{2}}\;{\left(e^{\frac{\theta x^{2}}{2}}-1\right)}^{\beta -1}\;e^{-\alpha {\left(e^{\frac{\theta x^{2}}{2}}-1\right)}^{\beta }}\alpha ,\;\theta ,\;\;\beta ,\;X>0.$$
- Generalized Rayleigh (GR) Distribution
  $$f(x)=2\alpha \gamma^{2}xe^{-{\left(\gamma x\right)}^{2}}{\left(1-e^{-{\left(\gamma x\right)}^{2}}\right)}^{\alpha -1}\;\alpha ,\;\gamma ,\;X\;>0.$$
- Two Parameter Rayleigh (TPR) Distribution
  $$f(x)=2\alpha (x-\mu )e^{-\alpha {\left(x-\mu \right)}^{2}}\;x>\mu ,\;\;\alpha >0.$$
- Modified Inverse Rayleigh Distribution.
  $$f(x)=(\alpha +\frac{2\theta }{x}){\left(\frac{1}{x}\right)}^{2}e^{-\frac{\alpha }{x}-\theta {\left(\frac{1}{x}\right)}^{2}}\;\alpha ,\;\theta ,\;X\;>0.$$
- Transmuted Inverse Rayleigh Distribution.
  $$f(x)=\frac{2\theta }{x^{3}}e^{\frac{-\theta }{x^{2}}}(1+\lambda -2\lambda e^{\frac{-\theta }{x^{2}}})\;\;\theta ,X\;>0.$$

#### Data set 1

**Patients receiving an analgesic.** The data set is taken from Gross and Clark [25] which consists of 20 observations of patients receiving an analgesic. The values are as follows

$$\begin{array}{llllllllll} 1.1 & 1.4 & 1.3 & 1.7 & 1.9 & 1.8 & 1.6 & 2.2 & 1.7 & 2.7\\ 4.1 & 1.8 & 1.5 & 1.2 & 1.4 & 3.0 & 1.7 & 2.3 & 1.6 & 2.0 \end{array}$$

Table 2 describes the parameter values of the probability models and also describes the goodness of fit measures. It is evident that the goodness of fit measures has fever values for the proposed model and hence it is concluded that the proposed model increased the flexibility of the model.

**Table 2:** Goodness of fit measures for data set 1.

Distribution	MLE			AIC	CAIC	BIC	HQIC	p-value
**APEIR**	**0.0041**	**0.7964**	**7.8595**	**37.2560**	**38.7560**	**40.2432**	**37.8391**	**0.1205**
EIR	0.8714	3.1686		46.3650	47.0709	48.3564	46.7537	0.1435
WR	11.8552	1.2364	0.0545	48.5149	50.0149	51.5021	49.0980	0.4597
GR	3.2748	0.6926		40.8050	41.5109	42.7965	41.1938	0.4630
TPR	0.6225	0.8352		39.6164	40.3223	41.6078	40.0051	0.3397

In Fig 4, the histogram represents the theoretical densities of the Alpha Power Exponentiated Inverse Rayleigh (APEIR), Two Parameter Rayleigh (TPR) and Exponentiated Inverse Rayleigh (EIR) by continuous red color line, dotted blue line and dotted green line respectively. It is evident from the above figure that the Alpha Power Exponentiated Inverse Rayleigh (APEIR) is leptokurtic and positively skewed as compared to other densities. Furthermore, the graph suggests that the Alpha Power Exponentiated Inverse Rayleigh (APEIR) distribution is less thick as compared to Two Parameter Rayleigh (TPR) distribution and thicker than Exponentiated Inverse Rayleigh (EIR) in the tail.

**Fig 3:**
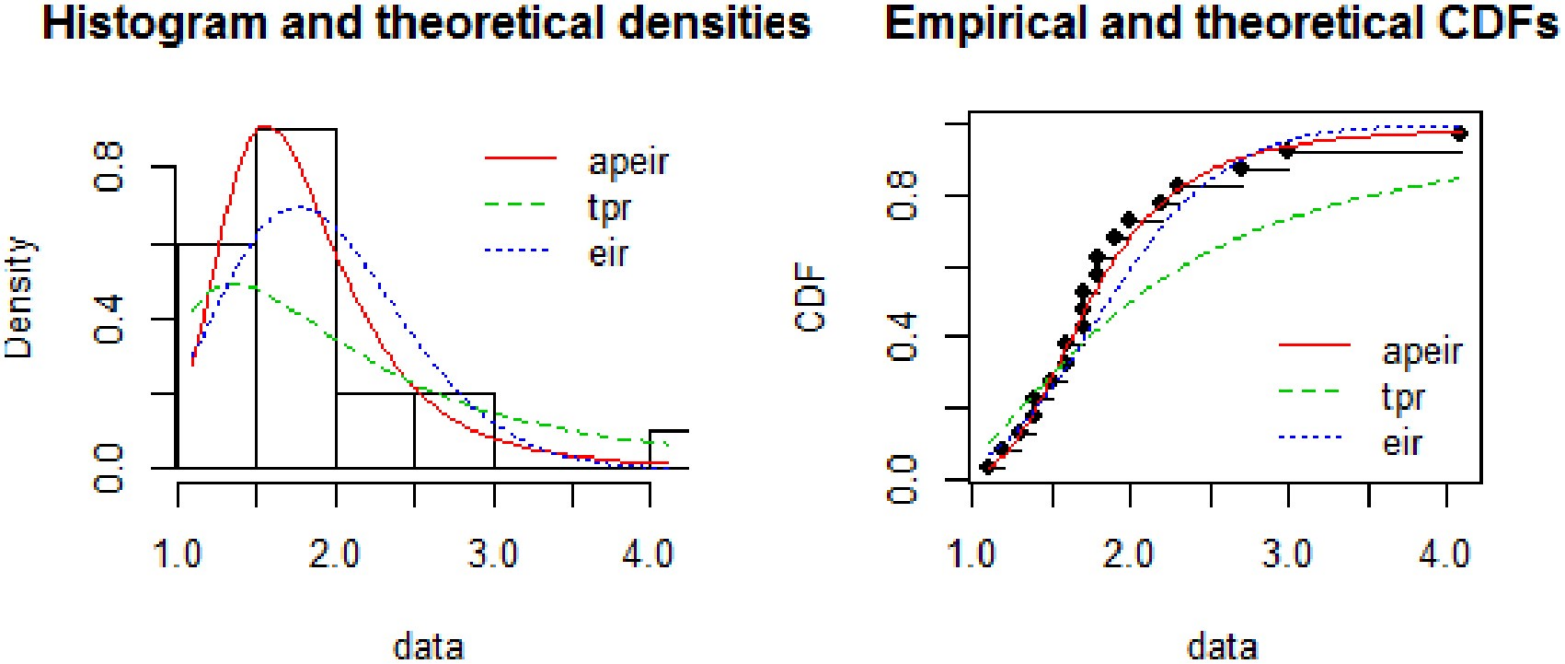
Comparison between fitted distributions for data set 1.

**Fig 4:**
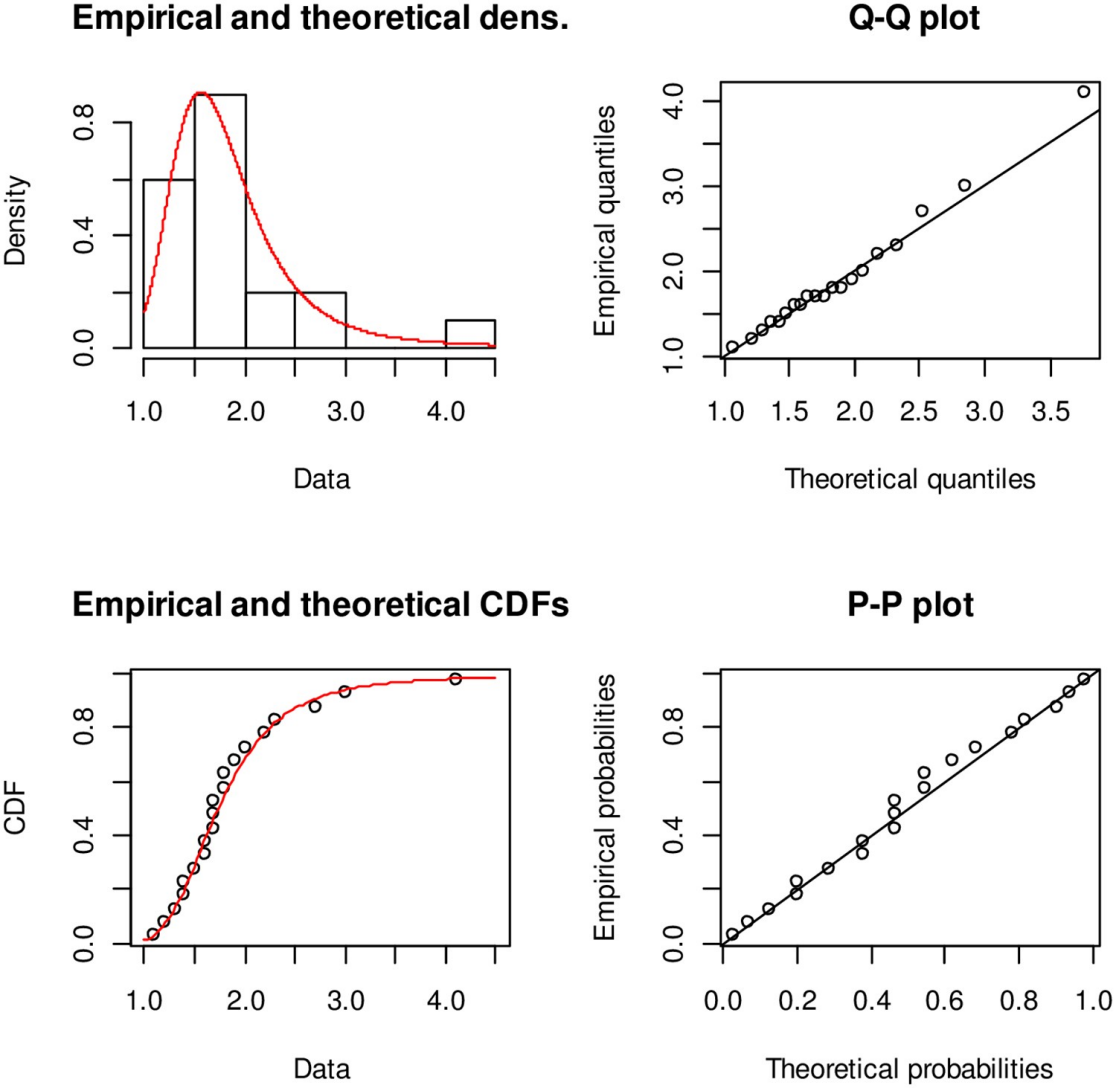
Probability density function, Q-Q plot, distribution function and P-P plot for data set 1.

If the plot of empirical against the theoretical CDFs is observed, then Alpha Power Exponentiated Inverse Rayleigh (APEIR) provides a better fit as compared to Exponentiated Inverse Rayleigh (EIR) and Two Parameter Rayleigh (TPR). Fig 3 describes the comparison of the proposed against other existing distributions. Fig 4 describes the PP-plot, QQ-plot, empirical and theoretical densities of Alpha Power Exponentiated Inverse Rayleigh (APEIR).

#### Data set 2

**Rainfall.** The second data set consists of thirty observations for the rainfall (in inches) of March in Minneapolis/St Paul [19]. The values are as follows

$$\begin{array}{lllllllllllllll} 0.77 & 1.74 & 0.81 & 1.20 & 1.95 & 1.20 & 0.47 & 1.43 & 3.37 & 2.20 & 3.00 & 3.09 & 1.51 & 2.10 & 0.52\\ 1.62 & 1.31 & 0.32 & 0.59 & 0.81 & 2.81 & 1.87 & 1.18 & 1.35 & 4.75 & 2.48 & 0.96 & 1.89 & 0.90 & 2.05 \end{array}$$

Table 3 describes the MLE of the probability models and describe the goodness of fit measures. Again, it is concluded that by increasing another parameter, we get a more significant result as compared to others.

**Table 3:** Goodness of fit measures for data set 2.

Distribution	MLE			AIC	CAIC	BIC	HQIC	p-value
**APEIR**	**13.7590**	**7.8802**	**0.0585**	**87.1186**	**88.0417**	**91.3222**	**88.4634**	**0.1031**
EIR	0.7668	1.1201		92.2730	92.7175	95.0754	93.1695	0.0638
TIR	0.6306	0.6674		88.2024	88.6469	91.0048	89.0989	0.2779
MIR	0.36016	0.5895		91.2599	91.7044	94.0624	92.15651	0.2698

**Fig 5:**
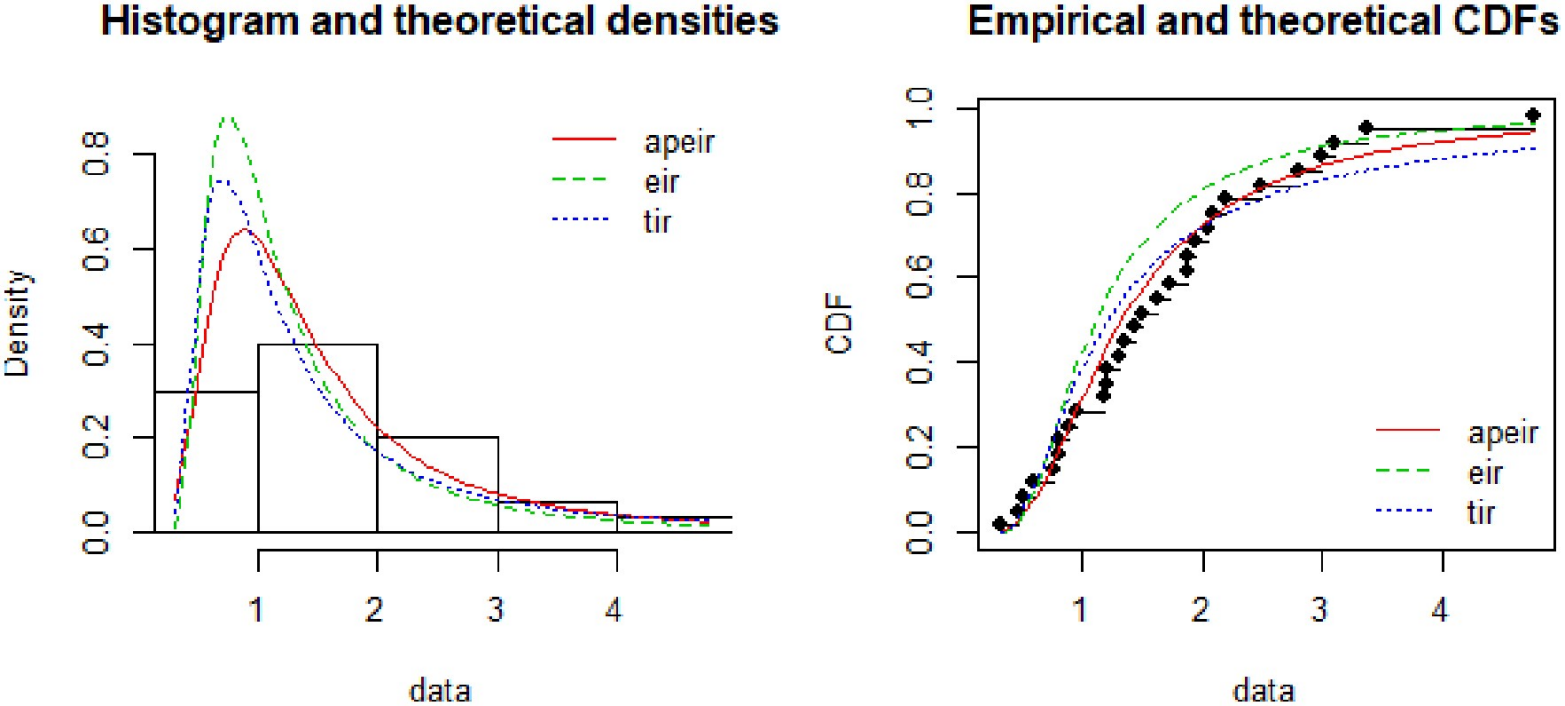
Comparison between fitted distributions for data set 2.

**Fig 6:**
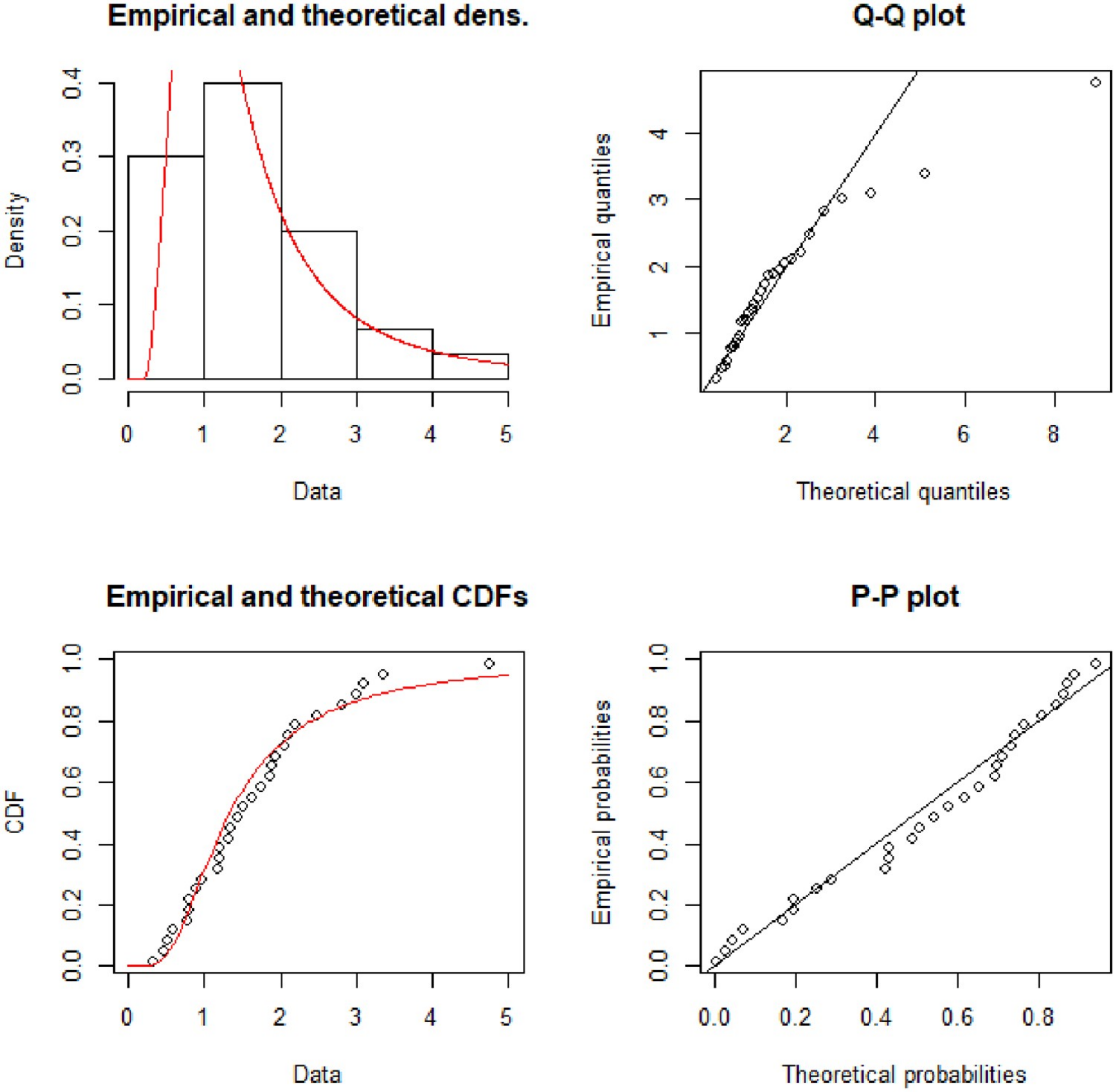
Probability density function, Q-Q plot, distribution function and P-P plot for data set 2.

Fig 5 describe the theoretical densities of Alpha Power Exponentiated Inverse Rayleigh (APEIR), Transmuted Inverse Rayleigh (TIR) and Exponentiated Inverse Rayleigh (EIR) by continuous red color line, dotted blue line and dotted green line respectively. Fig 5 clarify that Alpha Power Exponentiated Inverse Rayleigh (APEIR) is positively skewed. Moreover, the empirical and theoretical densities demonstrate that the Alpha Power Exponentiated Inverse Rayleigh (APEIR) provides a better fit to this data. Fig 6 describes the PP-plot, QQ-plot, empirical and theoretical densities of Alpha Power Exponentiated Inverse Rayleigh (APEIR).

## Conclusion

The paper presents a new probability distribution called Alpha Power Exponentiated Inverse Rayleigh (APEIR) distribution. The objective of the proposed distribution is to model the data with both monotonic and non-monotonic hazard rate shapes. The proposed distribution is of keen interest due its desirable properties. To estimate the parameters of the new distribution, Maximum likelihood estimation procedure is used. Furthermore, to evaluate the performance of the proposed distribution, it was fitted to two real data sets. The results showed that the new distribution provides a better fit to these data sets as compared to other versions of the Rayleigh distributions. Future researchers may derive new flexible distributions by using transmutation technique, or by increasing the scale or shape parameter to the proposed distributions in this paper. Further one can study the Bayesian analysis by choosing informative and non-informative priors.

## Supporting information

S1 Data Patients receiving an analgesic [22]. (TIF)

S2 Data Rainfall [19]. (TIF)
