# Supplementary figures and images for: Alpha-Power Exponentiated Inverse Rayleigh distribution and its applications to real and simulated data
Source: PLoS One. 2021 Jan 14;16(1):e0245253. doi: 10.1371/journal.pone.0245253 (PMC7808587; doi:10.1371/journal.pone.0245253)

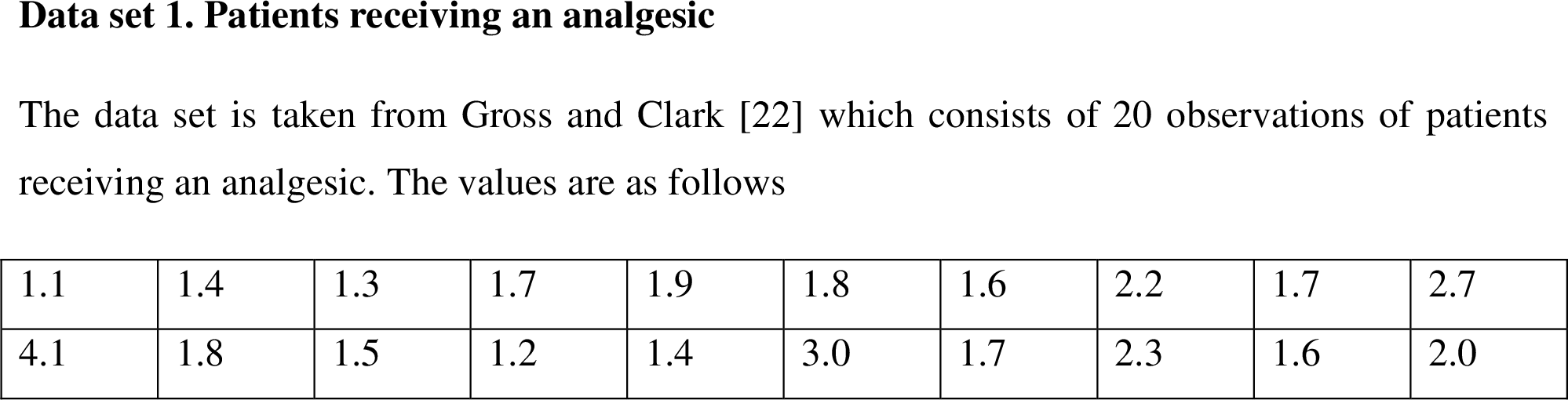

Supplement: S1 Data — (TIF) [file pone.0245253.s001.tif]

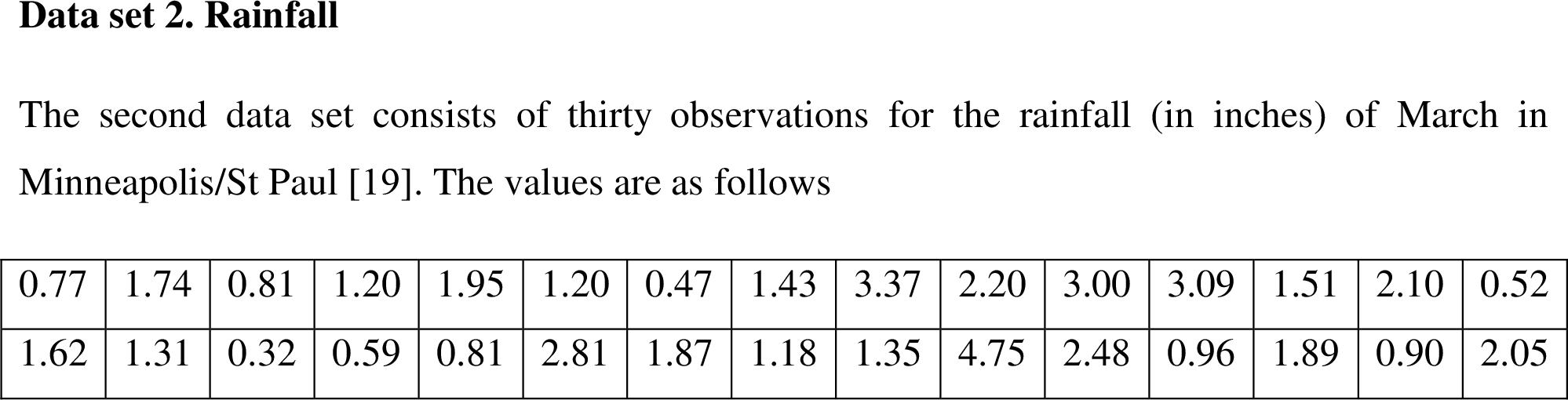

Supplement: S2 Data — (TIF) [file pone.0245253.s002.tif]
